# Supplementary material for: Mammal responses to global changes in human activity vary by trophic group and landscape
Source: Nat Ecol Evol. 2024 Mar 18;8(5):924–35. doi: 10.1038/s41559-024-02363-2 (PMC11090811; doi:10.1038/s41559-024-02363-2)
Supplement: Supplementary file 1 — Extended Results, Supplementary Figs. 1–4, Tables 1–10 and full Acknowledgements. [file 41559_2024_2363_MOESM1_ESM.pdf]

nature ecology & evolution

Article https://doi.org/10.1038/s41559-024-02363-2

Mammal responses to global changes in human activity vary by trophic group and landscape

In the format provided by the authors and unedited

Supplementary Information: Table of contents

Extended Results……………………………………………………………………………..2

Amount of animal activity…………………………………………………………2
Timing of animal activity……………………………………………………………3
Supplementary Fig. 1………………………………………………………………...4
Supplementary Fig. 2………………………………………………………………...5
Supplementary Fig. 3………………………………………………………………...6
Supplementary Fig. 4………………………………………………………………......7
Supplementary Table 1……………………………………………………………...8
Supplementary Table 2……………………………………………………………15
Supplementary Table 3……………………………………………………………16
Supplementary Table 4……………………………………………………………17
Supplementary Table 5……………………………………………………………18
Supplementary Table 6……………………………………………………………19
Supplementary Table 7……………………………………………………………20
Supplementary Table 8……………………………………………………………21
Supplementary Table 9……………………………………………………………22
Supplementary Table 10…………………………………………………………...23

Full Acknowledgements……………………………………………………………33

Extended Results

Amount of animal activity

Global model

The global model to explain variation in changes in the amount of animal activity contained 1065 population responses representing 102 unique projects and 163 species. The global model fixed effects accounted for 25.4% of the variance explained (pseudo-R^2). Trophic group was the strongest predictor of responses to higher human activity, with large herbivores showing the strongest increases in activity (+23%), and carnivores showing the strongest decreases (-10%; Fig. 2C; Supplementary Table 2; Supplementary Fig. 3). Animals in open habitats had reduced activity relative to animals in closed habitats (-15.8%, coefficient = -0.172; 95% CI = -0.3428 to -0.0018), and animal activity in developed areas (i.e., higher HMI; Table 1) generally increased with higher levels of human activity (+25%), while animals in less developed areas (lower HMI) decreased their activity (-6%, coefficient = 0.077; 95% CI = -0.001 to 0.156). We found no significant effects of diel activity, habitat breadth, diet breadth, lockdown stringency, or comparison type (year versus season) (Fig. 2C; Supplementary Fig. 3; Supplementary Table 2). The I^2 of the null model (a model without fixed effects) was 16.8%, highlighting that the random intercepts account for some heterogeneity in species responses to change in human activity. Of the random effects, 'project' accounted for 14.8%, suggesting that environmental context explains some of the variation in responses. 'family' only accounted for 2%, and 'species' 0%, of the variation, indicating that responses were highly heterogeneous within both families and species. We acknowledge that sample sizes were uneven across species and families (Supplementary Table 1), and that bias in estimates of these random effects is likely to be higher for species (and families) with fewer populations included in our sample.

Model selection of plausible interactions and non-linear terms

Of all of the plausible interactions and non-linear terms, an interaction between HMI and trophic group was the best supported (ΔAICc = -4.74 from the global model; Supplementary Table 3). The interaction was driven by the large omnivore trophic group having a negative relationship with HMI (Fig. 2D). Whereas large omnivore activity decreased by 50% from low to high HMI, activity of the other trophic groups increased by an average of 54% across this gradient (Fig. 2D).

Model selection on subsets of data

None of the models including hunting status improved on the global model, whether as an additive term or in plausible interactions with other effects (Supplementary Table 4). Similarly, including relative brain size or the magnitude of change in human activity (locally derived from camera trap detections) failed to improve model fit over the global model (Supplementary Table 5).

Timing of animal activity

Global model

The global model to explain variation in changes in the timing of animal activity contained 499 population responses representing 100 unique projects and 98 species. The global model fixed effects accounted for 30.2% of the variance explained (pseudo-R^2). The strongest predictor of changes in nocturnality was the degree of human landscape modification: as human activity increased, animals tended to become more nocturnal in more developed areas (+19.3%, Fig. 3C, D; Supplementary Fig 4; coefficient = 0.047; 95% CI = 0.026 to 0.069; Supplementary Table 6). Trophic group was also an important predictor of changes in nocturnality, with large carnivores becoming significantly more nocturnal than other groups (average +5.3% relative to other groups; Fig. 3C; Supplementary Table 6). Finally, the type of quasi-experimental comparison was an important predictor of changes in timing of animal activity, with comparisons between sampling periods in different years showing higher shifts to nocturnality relative to seasonal comparisons (+6.4%; Fig 3C; Supplementary Fig. 4; Supplementary Table 6). We found no significant effect of habitat breadth, diet breadth, diel activity, lockdown stringency or habitat openness on changes in animal nocturnality (Fig. 3C; Supplementary Fig. 4; Supplementary Table 6). The I^2 of the null model (a model without fixed effects) was 89.2%, highlighting that the random intercepts accounted for considerable heterogeneity in species responses to changes in human activity. Of the random effects, 'project' accounted for 79.0%, 'family' 0.9% and 'species' 9.3% of the variation, suggesting that environmental context explained the majority of variation in responses, with species explaining some, and responses being highly heterogeneous within families.

Model selection of plausible interactions and non-linear terms

We found strong support for an interaction between HMI and trophic group (ΔAICc= -10.23 from the global model). Most trophic groups had stronger increases in nocturnality along the disturbance gradient as human activity increased (mean +22.6%), whereas the increases in nocturnality for large carnivores did not change strongly with land-use disturbance (-4.2%, Fig. 3D; Supplementary Table 7).

Model selection on subsets of data

We found strong support for an interaction between population hunting status and HMI (ΔAICc= -3.83 from the global model; Supplementary Table 8) whereby hunted species showed stronger shifts to nocturnality (+26.6%) at higher human modification than their non-hunted counterparts (+13.5%, Fig 3E). All other interactions and non-linear effects did not improve on the global model (Supplementary Table 8). Furthermore, including locally derived change in human activity or relative brain size failed to improve model fit over the global model (Supplementary Table 9).

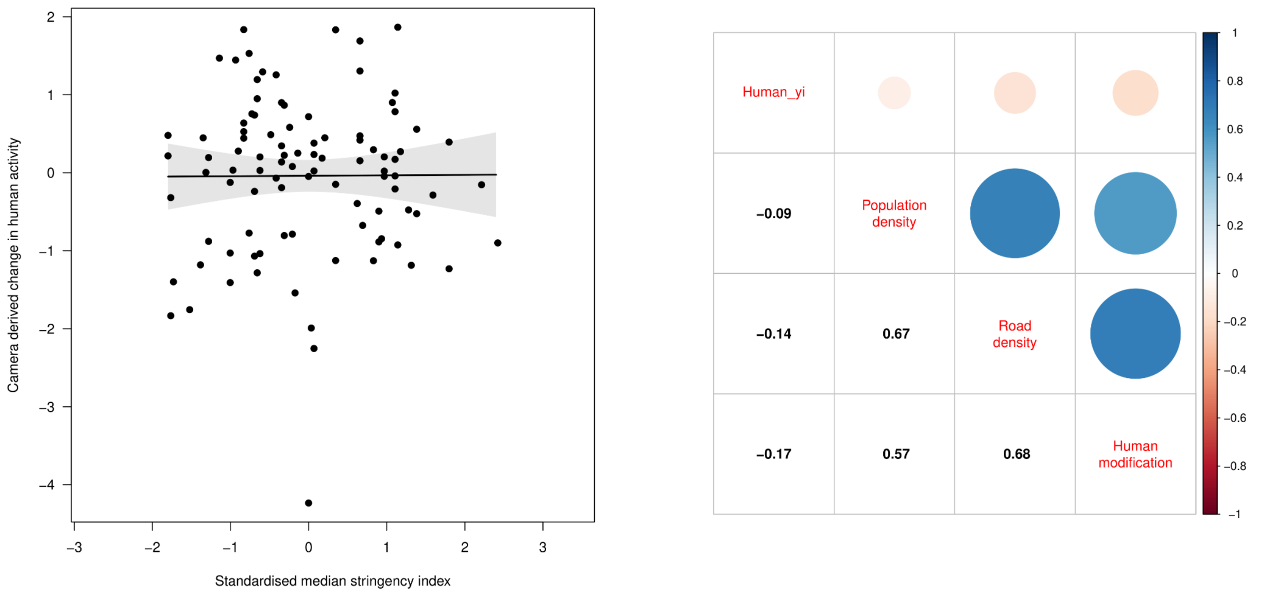

Supplementary Figure 1.

No relationship between project-level camera derived magnitude of change in human activity and COVID-19 lockdown stringency (left) and other metrics of human disturbance (right) for the subset of projects with empirical estimates of human activity. Black points = project-level mean estimates; black line = predicted relationship between stringency and local human-activity change derived from a linear model, grey polygon = 95% confidence interval of the linear model.

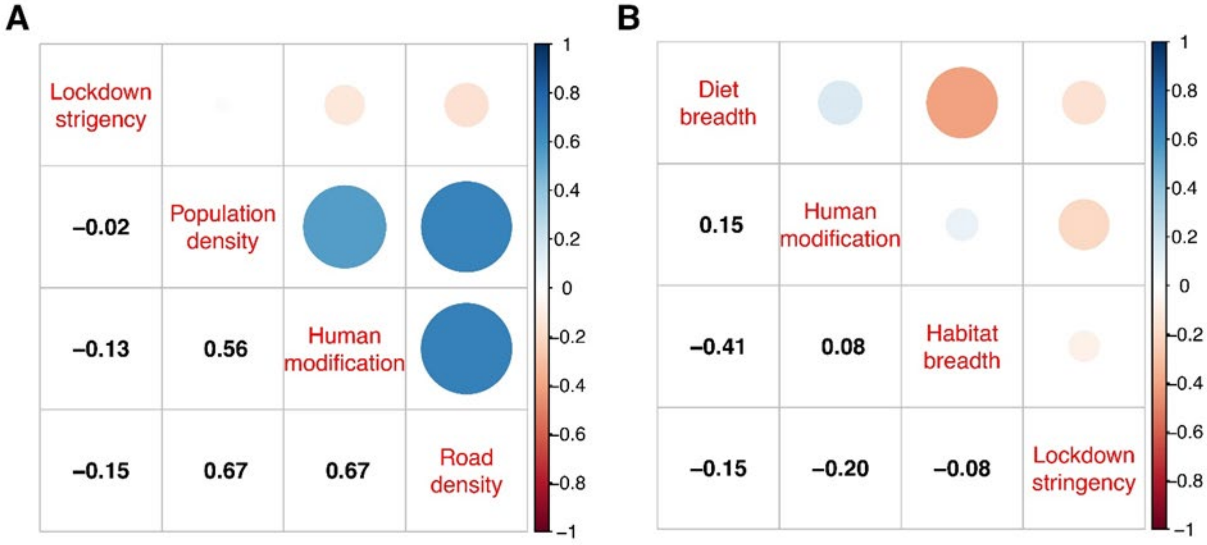

Supplementary Figure 2.

Correlation coefficients between putative predictor variables for all available data. (A) Project-level correlations between different metrics of human disturbance. (B) Population-level correlations between continuous predictors included in the meta-analysis models.

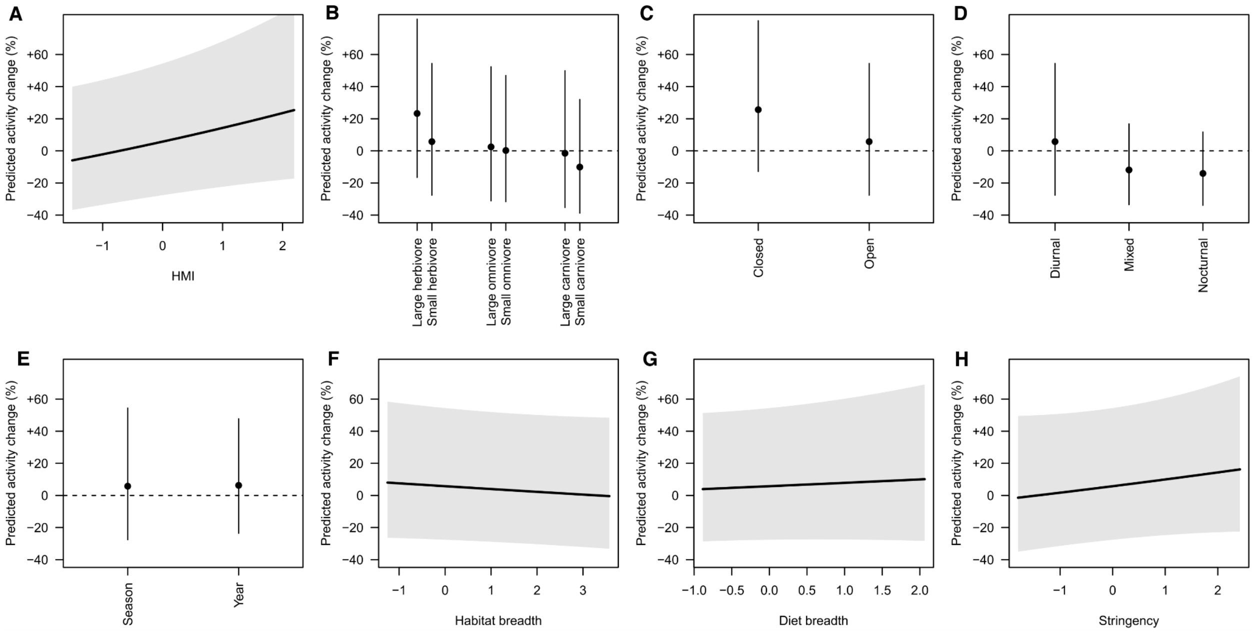

Supplementary Figure 3.

Model predictions for all of the variables included in the global model of changes in amount of animal activity. Where: black lines = model predictions for continuous variables; black points = model predictions for categorical variables; grey polygon/whiskers = 95% confidence interval for the predictions (n = 1065 project-species combinations from 102 independent projects).

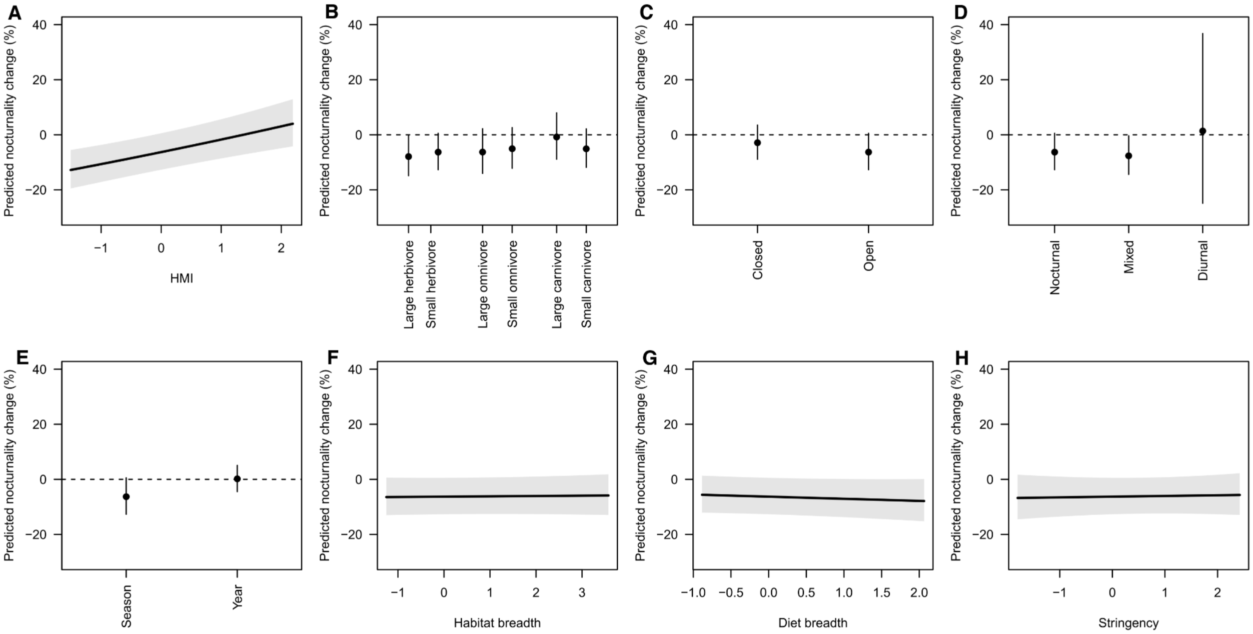

Supplementary Figure 4.

Model predictions for all of the variables included in the global model of changes in timing of animal activity. Where: black lines = model predictions for continuous variables; black points = model predictions for categorical variables; grey polygon/whiskers = 95% confidence interval for the predictions (n = 499 project-species combinations from 100 independent projects).

Supplementary Table 1.

List of species included in the analyses of responses to changes in the amount and timing of human activity, number of projects in which they were detected, standardized values of the traits used in the meta-analysis models, and ranges for human modification index (HMI) sampled and responses observed across populations for each analysis.

Species	# projects: amount	# projects: timing	Trophic group	Diet breadth	Activity cycle	Habitat breadth	Relative brain mass (g)	Human modification (min-max)	Amount response (min-max)	Timing response (min-max)
Acinonyx jubatus	1	NA	large_carnivore	-0.885	diurnal_only	0.557	0.4381	-1.03 to -1.03	-0.28 to -0.28	NA
Aepyceros melampus	2	2	large_herbivore	-0.885	cathemeral	-0.649	0.4744	-1.16 to -1.03	0.34 to 0.47	0.19 to 0.23
Alces alces	11	5	large_herbivore	-0.885	cathemeral	-0.649	0.4731	-1.51 to 1.57	-0.93 to 0.7	-1.21 to 0.3
Antilocapra americana	2	1	large_herbivore	-0.885	cathemeral	-0.046	0.4507	-1.3 to -1.25	-0.65 to 0.79	0.42 to 0.42
Atilax paludinosus	1	NA	small_carnivore	1.081	cathemeral	1.161	0.4178	-1.16 to -1.16	0.68 to 0.68	NA
Bassariscus astutus	1	NA	small_carnivore	0.098	nocturnal_only	0.557	0.4096	-0.87 to -0.87	-3.56 to -3.56	NA
Bison bison	3	2	large_herbivore	-0.885	cathemeral	0.557	0.4355	-1.45 to -0.54	0.06 to 1.1	-0.18 to 0.3
Bos javanicus	1	NA	large_herbivore	-0.885	cathemeral	-0.649	0.4531	-0.86 to -0.86	-0.46 to -0.46	NA
Canis adustus	1	1	small_carnivore	2.064	nocturnal_only	0.557	0.4315	-1.03 to -1.03	-0.64 to -0.64	0.05 to 0.05
Canis aureus	4	2	small_carnivore	-0.885	cathemeral	1.161	0.4644	-0.83 to 0.16	-0.13 to 2.56	-0.96 to -0.2
Canis latrans	65	39	small_carnivore	0.098	cathemeral	1.161	0.4856	-1.51 to 2.19	-3.56 to 2.05	-0.61 to 0.73
Canis lupus	25	8	large_carnivore	-0.885	cathemeral	2.367	0.4775	-1.51 to 1.24	-2.15 to 2.35	-1.6 to 0.22
Canis mesomelas	1	1	small_carnivore	1.081	cathemeral	2.367	0.4505	-1.03 to -1.03	-0.51 to -0.51	0.04 to 0.04
Canis rufus	1	1	large_carnivore	-0.885	cathemeral	0.557	0.4601	-1.45 to -1.45	-0.35 to -0.35	-0.12 to -0.12
Capreolus capreolus	22	20	large_herbivore	-0.885	cathemeral	0.557	0.4547	-1.03 to 1.46	-2.08 to 1.24	-0.71 to 0.41
Caracal caracal	1	NA	small_carnivore	-0.885	cathemeral	1.161	0.4244	-1.03 to -1.03	-1.57 to -1.57	NA
Castor canadensis	5	NA	small_herbivore	-0.885	cathemeral	-0.649	0.3850	-0.54 to 2.19	-0.69 to 2.04	NA
Castor fiber	3	NA	small_herbivore	-0.885	cathemeral	-0.649	0.3864	0.48 to 1.46	-2.92 to 0.92	NA
Catopuma badia	2	NA	small_carnivore	0.098	nocturnal_only	-1.253	0.4101	-0.86 to -0.85	-1.09 to 0.24	NA

Cephalophus natalensis	1	1	small_herbivore	0.098	nocturnal_only	-0.649	0.4680	-1.16 to -1.16	0.01 to 0.01	-0.12 to -0.12
Cervus elaphus	30	18	large_herbivore	-0.885	cathemeral	0.557	0.4897	-1.51 to 2.05	-2.08 to 2.22	-0.56 to 0.64
Cervus nippon	3	NA	large_herbivore	-0.885	cathemeral	0.557	0.4519	-0.65 to 1.23	0.5 to 3.44	NA
Chlorocebus pygerythrus	2	2	small_omnivore	1.081	diurnal_only	-0.046	0.4884	-1.16 to -1.03	0.07 to 0.11	-1.4 to -0.11
Civettictis civetta	1	1	small_carnivore	2.064	nocturnal_only	0.557	0.3846	-1.03 to -1.03	0.01 to 0.01	-0.02 to -0.02
Conepatus chinga	1	NA	small_carnivore	-0.885	nocturnal_only	-0.046	0.3573	-0.62 to -0.62	-0.69 to -0.69	NA
Conepatus leuconotus	1	NA	small_omnivore	-0.885	nocturnal_only	-0.046	0.3491	-0.87 to -0.87	1.3 to 1.3	NA
Connochaetes taurinus	2	1	large_herbivore	-0.885	cathemeral	-0.649	0.4951	-1.16 to -1.03	-0.78 to 1.72	-0.41 to -0.41
Crocuta crocuta	2	1	large_carnivore	-0.885	cathemeral	-0.649	0.455	-1.16 to -1.03	-0.5 to 0.69	-0.04 to -0.04
Cuniculus paca	2	2	small_herbivore	1.081	nocturnal_only	-1.253	0.3731	-1.44 to -1.19	-0.25 to 0.32	-0.04 to 0
Cynomys ludovicianus	1	1	small_herbivore	-0.885	diurnal_only	-0.046	0.2518	-1.25 to -1.25	-1.04 to -1.04	0.15 to 0.15
Dama dama	9	3	large_herbivore	-0.885	cathemeral	-0.046	0.4770	-0.83 to 1.24	-0.87 to 2.31	-0.01 to 1.29
Dasyprocta fuliginosa	1	1	small_herbivore	0.098	cathemeral	-1.253	0.3712	-1.44 to -1.44	-0.39 to -0.39	0.67 to 0.67
Dasyprocta punctata	1	1	small_herbivore	0.098	cathemeral	-0.046	0.3773	-1.19 to -1.19	0.6 to 0.6	0.1 to 0.1
Dasypus novemcinctus	15	7	small_carnivore	-0.885	nocturnal_only	1.161	0.2628	-1.44 to 1.46	-2.61 to 1.13	-0.3 to 0.1
Didelphis marsupialis	2	2	small_omnivore	2.064	nocturnal_only	-0.649	0.2620	-1.44 to -1.19	-0.26 to 0.15	0 to 0
Didelphis virginiana	41	19	small_omnivore	2.064	nocturnal_only	-0.046	0.2745	-1.45 to 2.05	-3.01 to 2.97	-0.13 to 0.09
Eira barbara	2	NA	small_carnivore	-0.885	cathemeral	-0.649	0.4369	-1.44 to -1.19	-1.04 to -0.21	NA
Elephas maximus	2	1	large_herbivore	-0.885	cathemeral	-0.046	0.5677	-0.86 to -0.85	-0.6 to 2.5	-0.69 to -0.69
Equus hemionus	1	NA	large_herbivore	-0.885	cathemeral	0.557	0.4990	0.16 to 0.16	0.13 to 0.13	NA
Equus quagga	2	2	large_herbivore	-0.885	cathemeral	-0.046	0.5057	-1.16 to -1.03	0.22 to 0.32	0.11 to 0.58
Erethizon dorsatum	15	1	small_herbivore	0.098	nocturnal_only	-0.046	0.355	-1.45 to 2.19	-2.61 to 2.03	-0.1 to -0.1
Felis silvestris	11	4	small_carnivore	-0.885	cathemeral	1.161	0.4265	-1.03 to 1.01	-1.86 to 1.77	-0.07 to 0.05
Galictis vittata	1	NA	small_carnivore	2.064	cathemeral	-0.649	0.4145	-1.19 to -1.19	-1.13 to -1.13	NA
Gazella dorcas	1	NA	large_herbivore	0.098	cathemeral	-1.253	0.3976	0.16 to 0.16	3.62 to 3.62	NA

Gazella gazella	1	NA	large_herbivore	-0.885	cathemeral	-0.046	0.4141	0.16 to 0.16	-1.66 to -1.66	NA
Giraffa camelopardalis	2	2	large_herbivore	-0.885	cathemeral	-0.046	0.4752	-1.16 to -1.03	-0.11 to 0.28	-0.17 to 0.18
Gulo gulo	5	1	small_carnivore	2.064	nocturnal_only	0.557	0.4648	-1.49 to -1.28	-1.09 to 2.02	0.45 to 0.45
Helarctos malayanus	3	NA	large_omnivore	1.081	nocturnal_only	-0.046	0.5491	-0.92 to -0.85	-0.71 to 0.61	NA
Hemigalus derbyanus	3	1	small_carnivore	-0.885	nocturnal_only	-1.253	0.3828	-0.92 to -0.85	-0.79 to 1.52	-0.01 to -0.01
Herpailurus yagouaroundi	1	NA	small_carnivore	-0.885	cathemeral	0.557	0.4226	-1.44 to -1.44	-1.44 to -1.44	NA
Herpestes brachyurus	2	NA	small_carnivore	1.081	cathemeral	-1.253	0.3493	-0.86 to -0.85	0.29 to 0.79	NA
Herpestes ichneumon	2	NA	small_carnivore	1.081	cathemeral	0.557	0.4014	-0.1 to 0.16	-2.51 to -1.37	NA
Herpestes semitorquatus	1	NA	small_carnivore	1.081	cathemeral	-1.253	0.3851	-0.86 to -0.86	0.7 to 0.7	NA
Hippopotamus amphibius	2	1	large_herbivore	-0.885	nocturnal_only	1.161	0.436	-1.16 to -1.03	-1.12 to -0.55	-0.03 to -0.03
Hippotragus niger	1	NA	large_herbivore	-0.885	diurnal_only	-0.649	0.4578	-1.03 to -1.03	0.75 to 0.75	NA
Hyaena hyaena	1	NA	large_omnivore	-0.885	cathemeral	-0.649	0.4415	0.16 to 0.16	1.99 to 1.99	NA
Hystrix africaeaustralis	2	2	small_herbivore	0.098	nocturnal_only	0.557	0.3757	-1.16 to -1.03	-1.27 to 0.32	0.04 to 0.09
Hystrix brachyura	2	NA	small_herbivore	0.098	nocturnal_only	-0.046	0.4076	-0.92 to -0.86	-0.51 to 1.56	NA
Hystrix crassispinis	2	1	small_herbivore	0.098	nocturnal_only	-1.253	0.3450	-0.86 to -0.85	-1.01 to 1.05	0.01 to 0.01
Hystrix cristata	2	2	small_herbivore	0.098	nocturnal_only	1.764	0.3847	-0.04 to 0.99	-0.08 to 0.12	-0.02 to 0.01
Hystrix indica	1	1	small_herbivore	0.098	nocturnal_only	0.557	0.3915	0.16 to 0.16	0.19 to 0.19	-0.03 to -0.03
Ichneumia albicauda	2	2	small_carnivore	-0.885	nocturnal_only	1.161	0.3979	-1.16 to -1.03	-0.57 to 0.19	-0.02 to -0.01
Kobus ellipsiprymnus	2	1	large_herbivore	-0.885	cathemeral	0.557	0.4762	-1.16 to -1.03	-1.4 to 0.16	0.03 to 0.03
Leopardus pardalis	2	2	small_carnivore	0.098	nocturnal_only	-0.046	0.4523	-1.44 to -1.19	-0.2 to 0.54	-0.01 to 0.16
Leopardus wiedii	1	NA	small_carnivore	0.098	cathemeral	-0.046	0.4264	-1.44 to -1.44	-0.76 to -0.76	NA
Lepus americanus	17	9	small_herbivore	-0.885	nocturnal_only	-0.649	0.3092	-1.51 to 1.57	-1.65 to 2.25	-0.06 to 0.26
Lepus californicus	5	1	small_herbivore	-0.885	nocturnal_only	1.161	0.3333	-1.25 to 0.17	-2.86 to -0.51	-0.18 to -0.18
Lepus capensis	1	NA	small_herbivore	-0.885	nocturnal_only	0.557	0.3089	0.16 to 0.16	2.11 to 2.11	NA
Lepus corsicanus	1	1	small_herbivore	-0.885	nocturnal_only	0.557	0.3181	0.99 to 0.99	0.61 to 0.61	-0.13 to -0.13

Lepus europaeus	23	14	small_herbivore	-0.885	nocturnal_only	-0.046	0.3200	-1.03 to 1.46	-1.54 to 3.43	-0.11 to 1.15
Lepus granatensis	1	1	small_herbivore	-0.885	nocturnal_only	0.557	0.3131	-0.1 to -0.1	-0.47 to -0.47	-0.06 to -0.06
Lepus timidus	1	NA	small_herbivore	-0.885	nocturnal_only	1.161	0.3386	0.58 to 0.58	2.16 to 2.16	NA
Lepus townsendii	4	1	small_herbivore	-0.885	nocturnal_only	-0.046	0.3213	-1.3 to 1.57	-3.12 to 0.23	-0.21 to -0.21
Lepus victoriae	2	2	small_herbivore	-0.885	nocturnal_only	-0.649	0.3113	-1.16 to -1.03	-0.49 to 0.12	0.01 to 0.08
Lontra canadensis	10	NA	small_carnivore	-0.885	cathemeral	1.161	0.4460	-1.49 to 1.58	-1.55 to 1.31	NA
Loxodonta africana	2	2	large_herbivore	-0.885	cathemeral	0.007	0.5590	-1.16 to -1.03	-0.31 to 0.26	-0.15 to 0.18
Lutra lutra	2	NA	small_carnivore	1.081	cathemeral	1.161	0.4249	-0.83 to 1.01	-1 to -0.78	NA
Lycalopex culpaeus	2	1	small_omnivore	-0.885	nocturnal_only	1.161	0.4197	-0.62 to 1.2	-0.68 to 1.04	0.25 to 0.25
Lycalopex griseus	2	2	small_omnivore	1.081	nocturnal_only	-0.046	0.4172	-0.62 to 1.2	-0.05 to 1.33	-0.19 to -0.1
Lycaon pictus	2	1	large_carnivore	-0.885	cathemeral	0.557	0.485	-1.16 to -1.03	-2.08 to -1	0.15 to 0.15
Lynx canadensis	5	4	small_carnivore	0.098	nocturnal_only	-0.649	0.4648	-1.51 to -1.28	-0.77 to 0.41	-0.27 to 0.09
Lynx lynx	8	2	large_carnivore	-0.885	nocturnal_only	1.161	0.4303	-1.03 to 1.23	-3.62 to 1.27	0.06 to 0.08
Lynx rufus	44	8	small_carnivore	-0.885	nocturnal_only	0.557	0.4481	-1.51 to 2.19	-2.78 to 2.94	-0.28 to 0.27
Macaca fascicularis	3	NA	small_carnivore	0.098	diurnal_only	-0.046	0.4968	-0.92 to -0.85	-1.38 to 3.54	NA
Macaca nemestrina	3	2	small_herbivore	-0.885	diurnal_only	-0.649	0.5264	-0.92 to -0.85	-0.21 to 1.13	-1.44 to 0.32
Marmota caligata	4	2	small_herbivore	1.081	diurnal_only	-0.649	0.3249	-1.51 to -1.45	-0.44 to 0.59	-0.02 to 0.22
Marmota monax	11	1	small_omnivore	1.081	diurnal_only	-0.046	0.3283	0.34 to 1.58	-1.55 to 2.73	0.61 to 0.61
Martes caurina	1	NA	small_carnivore	2	cathemeral	1	NA	-1.45 to -1.45	2.77 to 2.77	NA
Martes foina	13	6	small_carnivore	1.081	cathemeral	0.557	0.4140	-0.65 to 1.46	-3.58 to 1.77	-0.04 to 0.13
Martes martes	13	3	small_carnivore	0.098	nocturnal_only	-0.649	0.4236	-1.28 to 1.46	-2.15 to 1.66	-0.69 to -0.09
Mazama americana	2	2	large_herbivore	0.098	cathemeral	-1.253	0.4529	-1.44 to -1.19	-0.13 to 0.55	0.3 to 0.32
Mazama nemorivaga	1	NA	small_herbivore	1.081	cathemeral	-0.649	0.4293	-1.44 to -1.44	0.16 to 0.16	NA
Meles meles	23	13	small_omnivore	2.064	nocturnal_only	1.161	0.4250	-1.03 to 1.46	-2.82 to 3.01	-0.06 to 0.09
Mellivora capensis	2	1	small_omnivore	1.081	cathemeral	0.557	0.4728	-1.16 to -1.03	-0.66 to -0.52	-0.46 to -0.46

Mephitis mephitis	38	6	small_omnivore	2.064	nocturnal_only	0.557	0.3037	-1.51 to 2.19	-2.4 to 2.34	-0.14 to 0.16
Mungos mungo	2	NA	small_carnivore	0.098	cathemeral	-0.649	0.3339	-1.16 to -1.03	-0.87 to 1.59	NA
Muntiacus atherodes	2	1	small_herbivore	0.098	cathemeral	-0.649	0.4614	-0.92 to -0.86	-0.27 to -0.12	0.22 to 0.22
Muntiacus muntjak	3	1	small_herbivore	0.098	cathemeral	-0.649	0.4734	-0.92 to -0.85	0.1 to 1.57	-0.21 to -0.21
Mydaus javanensis	1	NA	small_carnivore	0.098	nocturnal_only	0.557	0.3841	-0.86 to -0.86	1.62 to 1.62	NA
Mydaus marchei	1	NA	small_carnivore	0.098	cathemeral	0.557	0.3495	-0.85 to -0.85	-1.87 to -1.87	NA
Myocastor coypus	2	NA	small_herbivore	-0.885	nocturnal_only	-0.649	0.3378	1.01 to 1.2	-0.63 to 1.21	NA
Myrmecophaga tridactyla	1	NA	large_carnivore	-0.885	cathemeral	1.161	0.4305	-1.44 to -1.44	-0.33 to -0.33	NA
Nasua narica	1	NA	small_omnivore	0.098	diurnal_only	-1.253	0.4603	-1.19 to -1.19	-0.77 to -0.77	NA
Neofelis diardi	1	NA	large_carnivore	-0.885	cathemeral	-1.253	0.4256	-0.86 to -0.86	-0.17 to -0.17	NA
Neofelis nebulosa	2	NA	small_carnivore	-0.885	cathemeral	-1.253	0.4318	-0.92 to -0.85	-0.07 to 0.98	NA
Nesotragus moschatus	1	NA	small_herbivore	0.098	cathemeral	-0.649	0.4017	-1.16 to -1.16	-1.63 to -1.63	NA
Nyctereutes procyonoides	2	NA	small_omnivore	1.081	nocturnal_only	0.557	0.4054	-0.65 to 0.06	-0.2 to 0.73	NA
Odocoileus hemionus	29	25	large_herbivore	-0.885	cathemeral	3.574	0.4638	-1.51 to 2.19	-0.98 to 1.27	-1.37 to 0.66
Odocoileus virginianus	53	51	large_herbivore	-0.885	cathemeral	3.574	0.4540	-1.51 to 1.67	-1.13 to 1.69	-1.51 to 0.65
Oreamnos americanus	4	1	large_herbivore	-0.885	cathemeral	0.557	0.4471	-1.51 to -1.4	-0.85 to 2.59	-0.36 to -0.36
Oreotragus oreotragus	1	NA	small_herbivore	-0.885	cathemeral	-0.046	0.4296	-1.03 to -1.03	0.14 to 0.14	NA
Orycteropus afer	1	NA	large_carnivore	-0.885	cathemeral	0.557	0.4252	-1.03 to -1.03	1.93 to 1.93	NA
Oryctolagus cuniculus	7	3	small_herbivore	-0.885	nocturnal_only	0.557	0.3100	-1.03 to 1.24	-1.76 to 1.05	-0.34 to 0.14
Ovis ammon	1	1	large_herbivore	-0.885	cathemeral	0.557	0.3925	0.3 to 0.3	-0.14 to -0.14	0.18 to 0.18
Ovis canadensis	4	1	large_herbivore	-0.885	cathemeral	-0.649	0.4606	-1.45 to -0.25	-1.06 to 2.76	-0.79 to -0.79
Ovis orientalis	1	NA	large_herbivore	-0.885	diurnal_only	-0.649	NA	0.06 to 0.06	-1.62 to -1.62	NA
Paguma larvata	1	NA	small_omnivore	1.081	nocturnal_only	-0.046	0.4113	-0.92 to -0.92	-0.28 to -0.28	NA
Panthera leo	2	2	large_carnivore	-0.885	cathemeral	0.557	0.459	-1.16 to -1.03	-0.22 to 0.21	-0.08 to 0
Panthera onca	2	1	large_carnivore	-0.885	cathemeral	1.764	0.447	-1.44 to -1.19	-0.46 to -0.22	-0.19 to -0.19

Panthera pardus	2	2	large_carnivore	-0.885	nocturnal_only	1.764	0.4502	-1.16 to -1.03	-0.52 to 0	-0.08 to -0.03
Papio ursinus	1	1	small_herbivore	1.081	diurnal_only	1.764	0.5392	-1.03 to -1.03	0.56 to 0.56	-0.54 to -0.54
Pardofelis marmorata	3	NA	large_carnivore	-0.885	nocturnal_only	-1.253	0.4370	-0.92 to -0.85	-1.09 to 2.03	NA
Pecari tajacu	3	2	large_omnivore	0.098	cathemeral	1.161	0.4633	-1.44 to 0.17	0.03 to 1.41	0.28 to 0.46
Pekania pennanti	10	NA	small_carnivore	2	cathemeral	1	NA	-1.27 to 1.45	-3.08 to 0.67	NA
Phacochoerus africanus	2	2	large_omnivore	-0.885	diurnal_only	-0.046	0.4278	-1.16 to -1.03	-0.63 to -0.37	0.33 to 0.81
Potamochoerus larvatus	1	NA	large_omnivore	0.098	nocturnal_only	-0.649	0.4495	-1.16 to -1.16	1.08 to 1.08	NA
Priodontes maximus	1	NA	large_carnivore	-0.885	nocturnal_only	-0.046	0.4224	-1.44 to -1.44	0.35 to 0.35	NA
Prionailurus bengalensis	3	NA	small_carnivore	-0.885	cathemeral	-0.046	0.4100	-0.92 to -0.85	-0.96 to 1.11	NA
Procyon lotor	58	37	small_omnivore	2.064	nocturnal_only	-1.253	0.4371	-1.45 to 2.19	-2.07 to 2.37	-0.24 to 0.17
Pteronura brasiliensis	1	NA	large_carnivore	0.098	diurnal_only	-0.046	0.4486	-1.44 to -1.44	-0.76 to -0.76	NA
Pudu puda	2	NA	small_herbivore	-0.885	cathemeral	-1.253	0.4516	-0.62 to 1.2	-0.75 to 2.41	NA
Puma concolor	19	5	large_carnivore	-0.885	cathemeral	1.161	0.4536	-1.51 to 0.3	-1.81 to 1.77	0 to 0.19
Raphicerus campestris	1	1	small_herbivore	-0.885	cathemeral	-0.046	0.4200	-1.03 to -1.03	0.23 to 0.23	-0.12 to -0.12
Redunca arundinum	2	NA	large_herbivore	-0.885	cathemeral	0.557	0.4492	-1.16 to -1.03	-2.26 to -1.8	NA
Rhynchogale melleri	1	NA	small_carnivore	1.081	nocturnal_only	-0.046	0.3715	-1.03 to -1.03	2.26 to 2.26	NA
Rupicapra rupicapra	6	2	large_herbivore	-0.885	cathemeral	0.557	0.4553	-1.03 to 0.84	-0.51 to 1.92	-0.2 to 0.54
Rusa unicolor	2	2	large_herbivore	-0.885	cathemeral	1.764	0.4669	-0.86 to -0.85	-0.19 to 0.7	-0.72 to 0.39
Smutsia temminckii	1	NA	small_carnivore	-0.885	nocturnal_only	-0.046	0.414	-1.03 to -1.03	-0.62 to -0.62	NA
Speothos venaticus	1	NA	small_carnivore	-0.885	cathemeral	1.161	0.4294	-1.44 to -1.44	0.63 to 0.63	NA
Sus barbatus	3	3	large_omnivore	-0.885	cathemeral	-1.253	0.4349	-0.92 to -0.85	-0.47 to 0.44	-0.72 to 1.84
Sus scrofa	34	20	large_omnivore	-0.885	cathemeral	2.367	0.4343	-1.09 to 1.24	-4.94 to 2.6	-0.5 to 0.44
Sylvicapra grimmia	2	2	small_herbivore	0.098	nocturnal_only	1.764	0.4416	-1.16 to -1.03	-0.54 to 0.41	-0.02 to 0.08
Sylvilagus aquaticus	1	NA	small_herbivore	-0.885	cathemeral	0.557	0.2983	1.46 to 1.46	0.92 to 0.92	NA
Sylvilagus floridanus	37	12	small_herbivore	-0.885	cathemeral	3.574	0.2860	-1.45 to 2.05	-4.51 to 2.52	-0.31 to 0.43

Sylvilagus palustris	1	NA	small_herbivore	-0.885	cathemeral	1.161	0.2734	-1.45 to -1.45	3.04 to 3.04	NA
Syncerus caffer	2	1	large_herbivore	-0.885	cathemeral	1.161	0.4760	-1.16 to -1.03	-0.86 to -0.72	0.82 to 0.82
Tamandua mexicana	1	NA	small_carnivore	-0.885	cathemeral	-0.649	0.4159	-1.19 to -1.19	1.53 to 1.53	NA
Tamandua tetradactyla	1	NA	small_carnivore	-0.885	cathemeral	-0.046	0.3903	-1.44 to -1.44	-0.06 to -0.06	NA
Tapirus bairdii	1	1	large_herbivore	-0.885	nocturnal_only	0.557	0.3809	-1.19 to -1.19	-0.13 to -0.13	-0.01 to -0.01
Tapirus terrestris	1	NA	large_herbivore	-0.885	nocturnal_only	1.161	0.4216	-1.44 to -1.44	0.07 to 0.07	NA
Taxidea taxus	12	NA	small_carnivore	0.098	nocturnal_only	-0.649	0.4429	-1.51 to 1.47	-2.34 to 1.42	NA
Tayassu pecari	1	NA	large_omnivore	1.081	cathemeral	0.557	0.4806	-1.19 to -1.19	1.64 to 1.64	NA
Tragelaphus angasii	2	2	large_herbivore	-0.885	cathemeral	-0.046	0.4709	-1.16 to -1.03	0.09 to 0.46	0.12 to 0.18
Tragelaphus scriptus	2	1	large_herbivore	-0.885	cathemeral	-0.649	0.4697	-1.16 to -1.03	-0.95 to 0.73	0.06 to 0.06
Tragelaphus strepsiceros	2	2	large_herbivore	-0.885	cathemeral	-0.046	0.4855	-1.16 to -1.03	-0.69 to 0.76	-0.6 to -0.1
Tragulus javanicus	1	1	small_herbivore	0.098	nocturnal_only	-0.649	0.3699	-0.92 to -0.92	-0.24 to -0.24	-0.61 to -0.61
Tragulus napu	2	1	small_herbivore	0.098	nocturnal_only	-0.649	0.3528	-0.92 to -0.85	0.43 to 0.54	-0.07 to -0.07
Trichys fasciculata	3	NA	small_herbivore	0.098	nocturnal_only	-1.253	0.3487	-0.92 to -0.85	-0.83 to 2.12	NA
Urocyon cinereoargenteus	24	4	small_omnivore	2.064	cathemeral	-0.649	0.4483	-1.27 to 1.47	-3.1 to 1.53	0 to 0.26
Ursus americanus	26	12	large_omnivore	0.098	cathemeral	0.557	0.4763	-1.51 to 1.99	-3.38 to 3.38	-1.5 to 0.73
Ursus arctos	14	9	large_omnivore	0.098	cathemeral	1.161	0.4748	-1.49 to 0.17	-1.28 to 1.25	-0.84 to 0.51
Viverra tangalunga	3	2	small_omnivore	0.098	cathemeral	-0.649	0.3645	-0.92 to -0.85	-0.45 to 1.14	-0.2 to 0.05
Vulpes vulpes	56	34	small_omnivore	0.098	cathemeral	1.764	0.4547	-1.45 to 2.19	-2.23 to 2.72	-0.19 to 0.63

Supplementary Table 2. Global model output for changes in amount of animal activity. Coefficient estimates from global model using change in the number of species detections as the response term. Test statistics and p-values derived from the default two-tailed Wald-type test using the *rmv.mv* function in the metafor R package (multiple comparisons were not performed). Significant contrasts shaded in dark grey ($p < 0.05$), marginal effects ($p < 0.10$) in light grey.

**Effect contrasts**	**Estimate**	**Standard error**	**Test statistic**	**P-value**
Intercept (comparison = season, trophic group = large herbivore, diel activity = diurnal, habitat = closed)	0.382	0.192	1.984	0.047
Comparison = year	0.005	0.110	0.044	0.965
Trophic group = large carnivore	-0.224	0.096	-2.334	0.020
Trophic group = large omnivore	-0.185	0.083	-2.222	0.026
Trophic group = small carnivore	-0.315	0.076	-4.133	0.000
Trophic group = small herbivore	-0.153	0.086	-1.780	0.075
Trophic group = small omnivore	-0.207	0.110	-1.881	0.060
Activity cycle = cathermeral	-0.182	0.151	-1.208	0.227
Activity cycle = nocturnal	-0.207	0.149	-1.388	0.165
Diet breadth	0.020	0.039	0.500	0.617
Habitat breadth	-0.017	0.020	-0.854	0.393
Stringency	0.039	0.039	0.988	0.323
HMI	0.078	0.040	1.934	0.053
Habitat closure = open	-0.172	0.087	-1.981	0.048

Supplementary Table 3. Model selection for interactions and non-linear effects in models of amount of animal activity. Where: ‘global model’ represents the additive model shown in Table S2; df = degrees of freedom; logLik = Log likelihood; AICc = Akaike information criterion corrected for small sample size; ΔAICc = change in AICc from the global model; wAIC = model weight. Models shaded in grey represent the best-supported model(s) discussed in the main text.

**Model terms**	**df**	**logLik**	**AICc**	**ΔAICc**	**wAICc**
Global model + HMI * Trophic group	22	-1459.8	2964.6	0.00	0.82
Global model	17	-1467.4	2969.4	4.74	0.08
Global model + Habitat closure * Trophic group	22	-1462.8	2970.5	5.88	0.04
Global model + HMI^2	18	-1467.2	2971.1	6.47	0.03
Global model + Habitat closure* HMI	18	-1467.3	2971.2	6.63	0.03

Supplementary Table 4. Model selection for effects of hunting on changes in amount of animal activity. Where: 'global model' represents the additive model shown in Supplementary Table 2; df = degrees of freedom; logLik = Log likelihood; AICc = Akaike information criterion corrected for small sample size; ΔAICc = change in AICc from the global model; wAICc = relative weight of support for the candidate model. Models shaded in grey represent the best-supported model(s) discussed in the main text.

**Model**	**df**	**AICc**	**ΔAICc**	**wAICc**
Global model	17	2701.23	0.00	0.327
Global model + hunting status	18	2701.24	0.01	0.326
Global model + hunting status * HMI	19	2701.98	0.75	0.225
Global model + hunting status * habitat closure	19	2703.27	2.04	0.118
Global model + hunting status * trophic group	23	2710.14	8.91	0.004

Supplementary Table 5. Model selection for effects of empirical magnitude of change in human activity (derived from camera traps), and relative brain size, on changes in amount of animal activity. Models shaded in grey represent the best-supported model(s) discussed in the main text. Where: 'global model' represents the additive model shown in Supplementary Table 2; df = degrees of freedom; logLik = Log likelihood; AICc = Akaike information criterion corrected for small sample size; ΔAICc = change in AICc from the global model; wAICc = relative weight of support for the candidate model.

**Model selection tables**	***df***	***AICc***	***ΔAICc***	***wAICc***
A) Locally derived human activity change				
Global model	*17*	2484.1	0.00	0.73
Global model + empirical human activity change	18	2486.1	2.01	0.27
B) Brain size				
Global model	17	2928.8	0.00	0.72
Global model + relative brain size	18	2930.7	1.92	0.28

Supplementary Table 6. Global model output for changes in timing of animal activity (nocturnality). Coefficient estimates are from the global model using the risk ratio for the proportion of nocturnal detections as the response term. Test statistics and p-values were derived from the default two-tailed Wald-type test using the *rmv.mv* function in the metafor R package (multiple comparisons were not performed). Significant contrasts are shaded in dark grey ($p < 0.05$).

Effect contrasts	Estimate	Standard error	Test statistic	P-value
Intercept (comparison = season, trophic group = large herbivore, diel activity = nocturnal, habitat = closed)	-0.047	0.037	-1.265	0.206
Comparison = year	0.067	0.031	2.123	0.034
Trophic group = large carnivore	0.074	0.025	2.993	0.003
Trophic group = large omnivore	0.018	0.027	0.644	0.520
Trophic group = small carnivore	0.030	0.023	1.331	0.183
Trophic group = small herbivore	0.017	0.022	0.794	0.427
Trophic group = small omnivore	0.031	0.031	0.996	0.319
Activity cycle = diurnal	0.079	0.149	0.529	0.597
Activity cycle = cathemeral	-0.014	0.016	-0.903	0.367
Diet breadth	-0.008	0.009	-0.875	0.382
Habitat breadth	0.001	0.006	0.226	0.821
Stringency	0.003	0.011	0.245	0.807
HMI	0.048	0.011	4.285	0.000
Habitat closure = open	-0.036	0.024	-1.496	0.135

Supplementary Table 7. Model selection for interactions and non-linear effects for animal nocturnality model. Where: 'global model' represents the additive model shown in Supplementary Table 6; df = degrees of freedom; logLik = Log likelihood; AICc = Akaike information criterion corrected for small sample size; ΔAICc = change in AICc from the global model; wAICc = relative weight of support for the candidate model. Models shaded in grey represent the best-supported model(s) discussed in the main text.

**Model**	**df**	**logLik**	**AICc**	**ΔAICc**	**wAICc**
Global model + HMI*Trophic group	22	-61.40	168.92	0.00	0.99
Global model + Habitat closure * Trophic group	22	-66.51	179.15	10.23	0.01
Global model + Habitat closure* HMI	18	-74.72	186.86	17.94	0.00
Global model	17	-76.66	188.60	19.67	0.00
Global model + HMI^2	18	-75.77	188.96	20.03	0.00

Supplementary Table S8. Model selection for effects of hunting on the animal nocturnality model. Where: ‘global model’ represents the additive model shown in Supplementary Table 6; df = degrees of freedom; logLik = Log likelihood; AICc = Akaike information criterion corrected for small sample size; ΔAICc = change in AICc from the global model; wAICc = relative weight of support for the candidate model. Models shaded in grey represent the best-supported model(s) discussed in the main text.

Model	df	AICc	ΔAICc	wAICc
Global + hunting status * HMI	19	246.7	0.00	0.75
Global	17	250.5	3.83	0.11
Global + hunting status	18	251.2	4.49	0.08
Global + hunting status * trophic group	23	253.0	6.30	0.03
Global + hunting status * habitat closure	19	253.1	6.38	0.03

Supplementary Table 9. Model selection for effects of empirical magnitude of change in human activity (derived from camera traps) and relative brain size on animal nocturnality. Where: df = degrees of freedom; AICc = Akaike information criterion corrected for small sample size; ΔAICc = change in AICc from the global model; wAICc = relative weight of support for the candidate model. Models shaded in grey represent the best-supported model(s) discussed in the main text.

**Model selection tables**	***df***	***AICc***	***ΔAICc***	***wAICc***
A) Locally derived human activity change				
Global model	17	149.2	0.00	0.75
Global model + empirical human activity change	18	151.3	2.16	0.25
B) Brain size				
Global model	17	188.6	0.00	0.74
Global model + Brain size	18	190.6	2.05	0.26

Supplementary Table 10. Details on camera trap projects and sampling periods included in the analyses of animal responses to changes in human activity.

Project Identifier	Contact	Country	Camera stations analyzed	Station spacing (km)	Bait use	Baited stations (%)	Period	Treatment Period	Comparison	Start	End	Duration (days)
2C2T	francesca.cagnacci@fmach.it	Italy	16	0.37	Yes	100	Control	High	Year	2019-03-09	2019-05-03	55
							Treatment	Low	Year	2020-03-09	2020-05-03	55
AL_Forest_Auburn	lepczyk@auburn.edu	USA	39	0.1	No	-	Treatment	High	Year	2020-10-11	2020-11-25	45
							Control	Low	Year	2019-10-12	2019-11-06	25
Allen Fort Hood	maxallen@illinois.edu	USA	59	0.92	Yes	66	Treatment	High	Year	2020-04-04	2020-05-06	32
							Control	Low	Year	2019-02-28	2019-04-08	39
AZ_Desert_McDowell_Sonoran_Preserve	helen@mcdowellsonoran.org	USA	18	0.79	No	-	Treatment	Low	Year	2020-09-10	2020-10-27	47
							Control	High	Year	2019-08-26	2019-10-21	56
Banff	jesse.whittington@canada.ca	Canada	300	2.67	No	-	Control	High	Year	2019-03-19	2019-05-19	61
							Treatment	Low	Year	2020-03-19	2020-05-19	61
BAPP	zaramcdonald@felidaefund.org	USA	24	1.77	No	-	Control	High	Season	2020-06-01	2020-08-15	75
							Treatment	Low	Season	2020-03-01	2020-05-15	75
BE_SPW_Gaume	valerie.dewaele@spw.wallonie.be	Belgium	98	1.02	No	-	Treatment	High	Year	2020-03-13	2020-06-13	92
							Control	Low	Year	2019-03-13	2019-06-13	92
BFNP_01	marco.heurich@npv-bw.bayern.de	Germany	84	1.35	No	-	Treatment	Low	Season	2020-03-01	2020-04-30	60
							Control	High	Season	2020-05-01	2020-06-30	60
Bighorn	fisherj@uvic.ca	Canada	82	3.91	Yes	100	Treatment	High	Season	2020-06-01	2020-07-31	60
							Control	Low	Season	2019-10-01	2019-11-30	60
Brodie	jedediah.brodie@gmail.com	Malaysia	43	0.98	No	-	Treatment	High	Year	2020-05-29	2020-08-13	76
							Control	Low	Year	2019-05-29	2019-08-13	76

Cathedral	cole.burton@ubc.ca	Canada	39	1.55	No	-	Treatment	High	Year	2020-07-05	2020-09-12	69
							Control	Low	Year	2019-07-05	2019-09-12	69
CJC	sturnina@gmail.com	CostaRica	17	1.46	No	-	Control	High	Year	2019-03-13	2019-10-29	230
							Treatment	Low	Year	2020-03-13	2020-10-29	230
CO_GrasslandOak_USAFA	kelliekuhn@gmail.com	USA	46	0.36	No	-	Control	High	Year	2019-09-03	2019-11-08	66
							Treatment	Low	Year	2020-08-30	2020-10-22	53
CT_Forest_Nye_Holman	miranda.l.davis@uconn.edu	USA	29	0.26	No	-	Control	High	Year	2019-09-02	2019-10-18	46
							Treatment	Low	Year	2020-09-03	2020-10-17	44
CT_Forest_Storrs	erin.kuprewicz@uconn.edu	USA	20	0.09	No	-	Treatment	Low	Year	2020-09-02	2020-10-26	54
							Control	High	Year	2019-09-01	2019-10-12	41
Danum_2020	mattluskin@gmail.com; z.amir@uq.net.au	Malaysia	32	0.43	No	-	Control	High	Season	2020-01-07	2020-03-16	69
							Treatment	Low	Season	2020-03-17	2020-05-26	70
DE_Forest_Wilmington	jacque.williamson@delaware.gov	USA	28	2.68	No	-	Treatment	High	Year	2020-08-21	2020-10-04	44
							Control	Low	Year	2019-08-27	2019-10-15	49
DKMP-PROTOKOL	erturk@kastamonu.edu.tr	Turkey	53	1.61	No	-	Treatment	High	Year	2020-05-30	2020-10-10	133
							Control	Low	Year	2019-05-30	2019-10-10	133
Edmonton	cstclair@ualberta.ca; catherine.shier@edmonton.ca; cjsteven@ualberta.ca	Canada	35	2.35	Yes	39	Treatment	High	Year	2020-04-17	2020-06-12	56
							Control	Low	Year	2019-04-17	2019-06-12	56
Foca	foca@ualberta.ca	Canada	65	1.9	No	-	Treatment	Low	Year	2020-05-01	2020-06-30	60
							Control	High	Year	2019-05-01	2019-06-30	60
GE	cole.burton@ubc.ca	Canada	35	1.17	No	-	Treatment	High	Year	2020-06-14	2020-07-31	47
							Control	Low	Year	2019-06-14	2019-07-31	47

Hamaarag LT monitoring	itai. mir@gmail.com	Israel	83	0.03	No	-	Control	High	Season	2020-09-18	2020-10-17	29
							Treatment	Low	Season	2020-03-17	2020-05-04	48
HI_Forest_O'ahu	pricemel@hawaii.edu	USA	18	0.18	No	-	Treatment	High	Year	2020-09-17	2020-10-20	33
							Control	Low	Year	2019-09-18	2019-11-01	44
IA_Anthropogenic_Ames	msrentz@iastate.edu	USA	47	0.21	No	-	Control	High	Year	2019-09-05	2019-11-02	58
							Treatment	Low	Year	2020-08-29	2020-11-01	64
IL_Forest_Urba	maxallen@illinois.edu	USA	26	0.23	No	-	Control	High	Year	2019-09-01	2019-09-30	29
							Treatment	Low	Year	2020-08-28	2020-09-30	33
IN_Forest_Purdue_Lugar	jmbrooke@purdue.edu	USA	14	0.48	No	-	Treatment	Low	Year	2020-09-03	2020-10-22	49
							Control	High	Year	2019-09-08	2019-11-07	60
IN_Forest_Purdue_PWA_Martell	jmbrooke@purdue.edu	USA	17	0.51	No	-	Control	Low	Year	2019-09-07	2019-10-29	52
							Treatment	High	Year	2020-09-01	2020-10-15	44
INBO_01	jim.casaer@inbo.be	Belgium	296	0.27	No	-	Treatment	High	Year	2020-03-13	2020-07-30	139
							Control	Low	Year	2019-03-13	2019-07-30	139
ISPRA_CP	barbara.franzetti@isprambiente.it	Italy	78	0.83	No	-	Control	High	Year	2019-03-30	2019-04-26	27
							Treatment	Low	Year	2020-05-08	2020-06-12	35
Kohl	michel.kohl@uga.edu	USA	50	11.52	Yes	44	Control	Low	Season	2020-02-24	2020-03-18	23
							Treatment	High	Season	2020-04-02	2020-04-25	23
Kootenays	emily.chow@gov.bc.ca	Canada	80	7.81	No	-	Treatment	High	Year	2020-03-15	2020-06-15	92
							Control	Low	Year	2019-03-15	2019-06-15	92
KS_Forest_Lawrence	rhagen@ku.edu	USA	25	0.33	No	-	Treatment	Low	Year	2020-09-22	2020-11-22	61
							Control	High	Year	2019-09-16	2019-10-27	41

LA_Forest_Hammond	teague.omara@gmail.com	USA	18	0.17	No	-	Treatment	High	Year	2020-09-02	2020-10-21	49
							Control	Low	Year	2019-09-11	2019-10-23	42
LC01	miroslav.kutal@hnutiduha.cz	Czech Republic	85	4.3	No	-	Treatment	High	Year	2020-03-16	2020-06-01	77
							Control	Low	Year	2019-03-16	2019-06-01	77
MA_Forest_Bridgewater_State_University	mfisherreid@bridgew.edu	USA	16	0.21	No	-	Treatment	High	Year	2020-08-30	2020-11-01	63
							Control	Low	Year	2019-08-29	2019-11-06	69
MalaMalaLondolozi	lucyksmyth@gmail.com	South Africa	95	1.71	No	-	Control	High	Year	2018-08-01	2018-09-24	54
							Treatment	Low	Year	2020-06-09	2020-08-03	55
MAMIRAUA	daniibarcelos@gmail.com	Brazil	26	1.95	No	-	Control	High	Year	2018-03-31	2018-05-31	61
							Treatment	Low	Year	2020-03-31	2020-05-31	61
Maremma	francesco.ferretti@unisi.it	Italy	53	0.73	No	-	Control	High	Year	2019-04-01	2019-06-15	75
							Treatment	Low	Year	2020-04-01	2020-06-15	75
MaxPlanck_01_Field	wikelski@ab.mpg.de	Germany	79	2.2	No	-	Control	High	Year	2018-07-01	2020-03-21	629
							Treatment	Low	Year	2020-03-22	2020-10-01	193
MaxPlanck_01_Forest	wikelski@ab.mpg.de	Germany	54	0.75	No	-	Control	High	Year	2018-07-01	2020-03-21	629
							Treatment	Low	Year	2020-03-22	2020-10-01	193
MaxPlanck_01_Yard	wikelski@ab.mpg.de	Germany	103	4.85	No	-	Treatment	High	Year	2020-03-22	2020-10-01	193
							Control	Low	Year	2018-07-01	2020-03-21	629
MELOCAM	pablo.ferreras@uclm.es	Spain	43	0.36	Yes	100	Treatment	Low	Season	2020-03-15	2020-06-21	98
							Control	High	Season	2020-06-22	2020-08-31	70
MI_Forest_Upper_Peninsula	truhubba@nmu.edu	USA	54	1.1	No	-	Treatment	High	Year	2020-09-03	2020-11-08	66
							Control	Low	Year	2019-08-29	2019-11-05	68

MK	cole.burton@ubc.ca	Canada	21	1.05	No	-	Treatment	High	Year	2020-07-11	2020-08-25	45
							Control	Low	Year	2019-07-11	2019-08-25	45
MO_Anthropogenic_University_of_Missouri	r.revord@missouri.edu	USA	29	0.35	No	-	Control	High	Year	2019-09-03	2019-10-24	51
							Treatment	Low	Year	2020-08-13	2020-11-02	81
MO_Forest_Bull_Shoals	spmaher@missouristate.edu	USA	19	0.45	No	-	Treatment	Low	Year	2020-08-29	2020-10-31	63
							Control	High	Year	2019-08-26	2019-11-02	68
Moreira2020	moreira.dario@gmail.com	Chile	6	1.88	No	-	Control	High	Season	2020-10-16	2021-01-10	68
							Treatment	Low	Season	2021-01-11	2021-02-07	86
MT_Grassland_SCBI	christopher3.hansen@umontana.edu	USA	101	0.63	No	-	Treatment	High	Year	2020-08-19	2020-10-15	57
							Control	Low	Year	2019-08-31	2019-09-23	23
MT_Roosevelt Ranch	McSheaW@si.edu	USA	40	0.94	No	-	Treatment	High	Year	2020-08-31	2020-11-01	62
							Control	Low	Year	2019-08-30	2019-11-07	69
NC_Dare County	mvcove@ncsu.edu	USA	20	2.28	No	-	Treatment	Low	Year	2020-09-25	2020-10-23	28
							Control	High	Year	2019-08-23	2019-11-05	74
NC_Schenck	rwkays@ncsu.edu	USA	29	0.28	No	-	Control	High	Year	2019-09-09	2019-10-05	26
							Treatment	Low	Year	2020-08-18	2020-09-30	43
NCDA	brett.furnas@wildlife.ca.gov	USA	22	26.2	No	-	Control	High	Season	2020-01-21	2020-03-20	59
							Treatment	Low	Season	2020-03-21	2020-05-21	61
ND_Grassland_Oakville_Prairie	susan.felege@und.edu	USA	14	0.53	No	-	Treatment	High	Year	2020-09-16	2020-11-04	49
							Control	Low	Year	2019-09-12	2019-10-18	36
NJ_Forest_Rutgers	lathrop@crssa.rutgers.edu	USA	13	0.22	No	-	Treatment	High	Year	2020-08-31	2020-11-04	65
							Control	Low	Year	2019-09-01	2019-11-01	61

NPHV	patrick.jansen@wur.nl	Netherlands	46	0.64	No	-	Treatment	Same	Year	2020-04-24	2020-05-15	21
							Control	Same	Year	2019-04-24	2019-05-15	21
NPTara_P1	dcirovic@bio.bg.ac.rs	Serbia	16	1.49	No	-	Control	High	Year	2019-03-01	2019-06-01	92
							Treatment	Low	Year	2020-03-01	2020-06-01	92
NY_Forest_Albany_Pine_Bush	dbogan@siena.edu	USA	29	0.23	No	-	Treatment	High	Year	2020-09-24	2020-11-20	57
							Control	Low	Year	2019-09-21	2019-10-24	33
NY_Forest_Dyken_Pond	dbogan@siena.edu	USA	28	0.21	No	-	Treatment	High	Year	2020-09-26	2020-10-29	33
							Control	Low	Year	2019-09-22	2019-10-25	33
NY_Forest_Mianus_River_Gorge	cmwnagy@gmail.com	USA	40	0.36	No	-	Treatment	High	Year	2020-09-29	2020-11-10	42
							Control	Low	Year	2019-09-05	2019-11-08	64
NY_Forest_Paul_Smiths_College	ccincotta@paulsmiths.edu	USA	12	0.16	No	-	Treatment	High	Year	2020-09-14	2020-11-06	53
							Control	Low	Year	2019-09-05	2019-10-15	40
NY_Forest_St_Lawrence_University	barthelmess@stlawu.edu	USA	26	2.5	No	-	Treatment	High	Year	2020-09-24	2020-11-09	46
							Control	Low	Year	2019-09-17	2019-11-17	61
OFP	ajmarsha@umich.edu	Indonesia	50	0.55	No	-	Treatment	High	Year	2020-03-17	2020-04-28	42
							Control	Low	Year	2019-03-17	2019-04-28	42
OH_Forest_Huston-Brumbaugh Nature Center	zornas@mountunion.edu	USA	18	0.29	No	-	Treatment	Low	Year	2020-08-24	2020-10-28	65
							Control	High	Year	2019-08-30	2019-11-01	63
OK_Grassland_Ardmore	slwebb@noble.org	USA	24	1.1	No	-	Treatment	Low	Year	2020-08-07	2020-10-12	66
							Control	High	Year	2019-09-23	2019-10-10	17
OK_Grassland_Burneyville	slwebb@noble.org	USA	21	0.56	No	-	Treatment	Low	Year	2020-09-21	2020-10-12	21
							Control	High	Year	2019-09-23	2019-10-10	17

OKO_LOG	julian.weber@oeko-log.com	Germany	7	24.81	No	-	Treatment	High	Year	2020-03-01	2020-06-01	92
							Control	Low	Year	2019-03-01	2019-06-01	92
OR_Forest_Oregon_State_University	cara.appel@oregonstate.edu	USA	27	0.48	No	-	Treatment	Same	Year	2020-09-04	2020-10-24	50
							Control	Same	Year	2019-09-06	2019-10-23	47
PacificRimArray	yuri.zharikov@canada.ca	Canada	108	1.14	No	-	Control	High	Year	2019-03-01	2019-05-31	91
							Treatment	Low	Year	2020-03-01	2020-05-31	91
PCA_Chile	christian.osorio@carnivorosaustrales.org	Chile	20	3.22	Yes	100	Control	High	Season	2020-01-01	2020-02-28	58
							Treatment	Low	Season	2020-03-01	2020-04-30	60
PNAB_CAM	francesco.rovero@unifi.it	Italy	30	1.57	No	-	Treatment	High	Year	2020-06-11	2020-07-15	34
							Control	Low	Year	2019-06-11	2019-07-15	34
RI_Forest_University_of_Rhode_Island	bgerber@uri.edu	USA	22	0.31	No	-	Treatment	High	Year	2020-09-20	2020-11-28	69
							Control	Low	Year	2019-09-16	2019-11-08	53
SantaCruz	maxallen@illinois.edu	USA	74	3.87	No	-	Control	Low	Year	2017-03-19	2017-05-30	72
							Treatment	High	Year	2020-03-19	2020-05-30	72
SC_Forest_Piedmont	djachow@clemson.edu	USA	13	1.5	No	-	Treatment	High	Year	2020-09-01	2020-10-31	60
							Control	Low	Year	2019-08-31	2019-11-09	70
SD_Grassland_Custer_County	jalston@uwyo.edu	USA	18	0.24	No	-	Treatment	Low	Year	2020-09-15	2020-11-12	58
							Control	High	Year	2019-09-15	2019-11-07	53
SD_Shelterbelt_and_Grassland_Brookings	robert.lonsinger@okstate.edu	USA	19	1.28	No	-	Treatment	High	Year	2020-09-03	2020-10-21	48
							Control	Low	Year	2019-09-10	2019-10-30	50
SeatoSkyMamMon	kim.dawe@gmail.com	Canada	31	0.8	No	-	Control	High	Year	2019-06-01	2019-09-21	112
							Treatment	Low	Year	2020-06-01	2020-09-21	112

Silva-Rodriguez2020	eduardo.silva@uach.cl	Chile	8	0.9	No	-	Control	High	Season	2020-09-22	2020-11-06	45
							Treatment	Low	Season	2020-11-07	2020-12-22	45
Slovenia	miha.krofel@gmail.com	Slovenia	144	1.96	No	-	Treatment	High	Season	2020-03-15	2020-04-11	27
							Control	Low	Season	2020-01-01	2020-03-12	71
SouthChilcotinMountains	Robin.Naidoo@wwfus.org	Canada	61	2.19	No	-	Treatment	High	Year	2020-07-01	2020-08-31	61
							Control	Low	Year	2019-07-01	2019-08-31	61
SUCP	katie.remine@zoo.org; Robert.Long@Zoo.org; jordanma@seattleu.edu	USA	22	3.54	Yes	100	Control	High	Season	2020-07-04	2020-08-04	31
							Treatment	Low	Season	2020-06-03	2020-07-03	30
Tembe	lucyksmyth@gmail.com	South Africa	32	0.07	No	-	Control	High	Year	2019-04-12	2019-05-14	32
							Treatment	Low	Year	2020-04-12	2020-05-14	32
TN_Forest_Cheatham	cb3552@msstate.edu	USA	25	0.48	No	-	Treatment	High	Year	2020-10-06	2020-11-16	41
							Control	Low	Year	2019-09-19	2019-11-04	46
TN_Forest_Cumberland Gap National Historical Park	laroy.brandt@lmunet.edu	USA	9	0.56	No	-	Treatment	Low	Year	2020-08-19	2020-11-03	76
							Control	High	Year	2019-09-06	2019-11-04	59
TX_Forest_Pineywoods_ Angeli	christopher.schalk@usda.gov	USA	29	0.24	No	-	Treatment	High	Year	2020-10-02	2020-10-26	24
							Control	Low	Year	2019-09-21	2019-10-11	20
TX_Grassland_Abilene	leet@acu.edu	USA	21	0.4	No	-	Treatment	High	Year	2020-08-18	2020-10-24	67
							Control	Low	Year	2019-09-01	2019-11-02	62
TX_Grassland_Matador	caroline.ellison@tpwd.texas.gov	USA	20	1.68	No	-	Treatment	High	Year	2020-08-31	2020-11-02	63
							Control	Low	Year	2019-09-05	2019-11-05	61
Umea	tim.hofmeester@slu.se	Sweden	12	8.43	No	-	Treatment	High	Season	2020-04-28	2020-06-13	46
							Control	Low	Season	2020-06-14	2020-07-30	46

UniBrno_01	r.plhal@seznam.cz	Czech Republic	28	0.34	No	-	Treatment	High	Season	2020-03-15	2020-04-07	23
							Control	Low	Season	2020-02-27	2020-03-14	16
UniHasselt_01	jim.casaer@inbo.be	Belgium	114	0.54	No	-	Control	High	Year	2019-04-01	2019-06-01	61
							Treatment	Low	Year	2020-04-01	2020-06-01	61
UniZagreb_Agri_01	-	Croatia	35	2.05	No	-	Treatment	Low	Year	2020-03-15	2020-04-23	39
							Control	Low	Year	2019-03-15	2019-04-23	39
UT_Desert_Wasatch	austin.m.green@utah.edu	USA	44	1.79	No	-	Treatment	High	Year	2020-09-05	2020-11-05	61
							Control	Low	Year	2019-09-01	2019-11-17	77
VA_Forest_Richmond(rural)	jsevin@richmond.edu	USA	16	0.22	No	-	Control	High	Year	2019-09-04	2019-11-11	68
							Treatment	Low	Year	2020-09-07	2020-11-03	57
VA_Forest_Richmond(suburban)	jsevin@richmond.edu	USA	12	0.33	No	-	Treatment	Low	Year	2020-09-13	2020-10-28	45
							Control	High	Year	2019-08-08	2019-11-06	90
VA_Forest_SCBI_GEO	McSheaW@si.edu	USA	61	0.13	No	-	Control	High	Year	2019-08-27	2019-10-09	43
							Treatment	Low	Year	2020-09-01	2020-10-30	59
Vinci	claude.miaud@cefe.cnrs.fr	France	64	16.57	No	-	Treatment	High	Year	2020-03-01	2020-06-01	92
							Control	Low	Year	2019-03-01	2019-06-01	92
WA_Forest_Seattle_Urban_Carnivores	jordanma@seattleu.edu	USA	19	3.03	No	-	Treatment	High	Year	2020-08-24	2020-11-21	89
							Control	Low	Year	2019-08-31	2019-11-08	69
Waterton Corridor	kimberly.pearson@ca da.ca	Canada	38	0.22	No	-	Control	High	Year	2019-03-15	2019-05-15	61
							Treatment	Low	Year	2020-03-15	2020-05-15	61
WI_Forest_Whitewater	romeroa@gmail.com	USA	13	0.22	No	-	Control	High	Year	2019-09-07	2019-11-01	55
							Treatment	Low	Year	2020-09-01	2020-10-21	50

WV_Forest_WVU_Research_Forest	christopher.rota@mail.wvu.edu	USA	44	0.36	No	-	Treatment	High	Year	2020-09-12	2020-11-06	55
							Control	Low	Year	2019-09-24	2019-10-24	30
WY_Grassland_Carbon_County	jalston@uwyo.edu	USA	20	0.35	No	-	Treatment	High	Year	2020-09-13	2020-11-07	55
							Control	Low	Year	2019-09-07	2019-11-10	64
YWW	dlaffert@nmu.edu; truhubba@nmu.edu	USA	30	1.17	No	-	Treatment	High	Season	2020-04-17	2020-08-01	106
							Control	Low	Season	2020-01-01	2020-04-11	101
LifeLynxCro_01	magda.sindicic@vef.unizg.hr	Croatia	49	5.44	No	-	Treatment	High	Year	2020-03-15	2020-05-15	61
							Control	Low	Year	2019-03-15	2019-05-15	61

Full Acknowledgements

We thank Mason Fidino, Mahdieh Tourani, and an anonymous referee for constructive feedback on earlier versions of the manuscript.

This paper was written and prepared in part by U.S. Government employees on official time, and therefore it is in the public domain and not subject to copyright.
This research was supported in part by the USDA Forest Service.
The findings and conclusions in this publication are those of the authors and should not be construed to represent an official USDA, Forest Service, or U.S. Government determination or policy.

Individual projects that contributed to this study received funding from the following:

British Columbia Ministry of Environment and Climate Change Strategy, BC Parks
Clark Stevens Endowed Professorship
James Lea Foundation
Wilburforce Foundation
Charlotte Martin Foundation
Pemberton Wildlife Association
National Science Centre, Poland (grant number 2017/25/B/NZ8/02466)
Fundación Naturaleza El Salvador
USFWS Federal Aid in Wildlife Restoration Grant to Wisconsin Dept. of Natural Resources
Idea Wild
Rufford Small Grants
Sea Turtle Conservancy
Upper Delta Region Biodiversity Scholarship, National Science Foundation (Award # 1564954)
Watershed Studies Institute, Murray State University
Lincoln Memorial University
Cumberland Mountain Research Center
FCT/MCTES to CESAM (UIDP/50017/2020+UIDB/50017/2020+ LA/P/0094/2020)
This work makes use of data and/or infrastructure provided by the Research Institute for Nature and Forests (INBO) and funded by Research Foundation - Flanders (FWO) as part of the Belgian contribution to LifeWatch
Fish and Wildlife Compensation Program
Pittman-Robertson Wildlife Restoration Act
Public Service of Wallonia
Regional Government of Castilla-La Mancha and the European Regional Development Fund (ERDF) (Project SBPLY/17/180501/000184)
National Science Centre, Poland (grant number 2017/25/B/NZ8/02466)
University of North Dakota Biology Department
General Directorate of Nature Conservation and National Parks
Maremma Regional Park Agency
German Society for Mammalian Biology - DGS Project Funding 2018
Forest Resource Improvement Association of Alberta
InnoTech Alberta

Alberta Environment and Parks
Yellowstone to Yukon Initiative
Canadian Mountain Network
Mitacs Canada
LIFE Lynx LIFE16 NAT/SI/000634
Alberta Conservation Association
Northern Alberta Chapter of Safari Club International
Alberta Fish and Game Association
Beaver Hills Initiative
Friends of Elk Island Society
Segretariato Generale per la Presidenza della Repubblica
Servizio Tenuta presidenziale di Castelporziano
Parks Victoria
Global Change and Sustainability Center - University of Utah
University of Kansas Environmental Studies Program
Boone and Crockett Club
University of Montana Boone and Crockett Wildlife Conservation Program
National Science Foundation (NSF) LTREB awards #2038704 and 1556248 to M.H.
Parks Canada
Urban Futures platform - Swedish University of Agricultural Sciences
South Carolina Department of Natural Resources
Conselho de Aperfeiçoamento de Pessoal de Nível Superior (CAPES, proc. BEX 1 298/2015-01)
German Federal Ministry of Education and Research (BMBF) Collaborative Knowledge Transfer Project 'WTimpact' (funding number 01IO1725)
Slovenian Research and Innovation Agency (ARRS, grants no. N1–0163 and J1-50013)
EuroNatur Foundation, European Union's Interreg Danube Transnational Programme (SaveGREEN DTP3-314-2.3)
New Jersey Agricultural Experiment Station
USDA Forest Service, Pacific Northwest Research Station
South Dakota Agricultural Experiment Station
Woodland Park Zoo
Seattle University
The Royal Commission for AlUla
South African National Biodiversity Institute
Singita
University of California, Davis
University of Michigan
Victoria University of Wellington
Chinese Academy of Sciences
Xishuang- banna Tropical Botanical Garden
Hellman Foundation
Leakey Foundation
Orangutan Conservancy
Fulbright Foundation
Mohamed bin Zayed Species Conservation Fund
AZA Ape TAG Initiative.

Vinci Autoroute, contract VINCI-CNRS
Grant ANID PIA/BASAL FB210006
Tyax Adventures
Servizio Faunistico, Provincia Autonoma di Trento
Ministero per la Transizione Ecologica, Provincia Autonoma di Trento
Specific University Research Fund MENDELU (IGA-LDF22TP2-107)
Fundación Naturaleza El Salvador
Conselho Nacional de Desenvolvimento Científico e Tecnológico (CNPq)
Carnivora Dinarica Interreg project
McIntire-Stennis WVA00818
McDowell Sonoran Conservancy
The Gordon and Betty Moore Foundation (GBMF9881 to C.R.) and the National Geographic Society (NGS-82515R-20 to C.R.) to the COVID-19 Bio-Logging Initiative
Segre Foundation
Sigrid Rausing Trust
H. Batubay Özkan
Barbara Watkins
John and Adrienne Mars
City of Edmonton
University of Alberta (Faculty of Science Research Fellowship and Teaching Assistantship)
Natural Sciences and Engineering Research Council of Canada (NSERC; Discovery Grant; RGPIN-2017-05915)
NSERC Canada Graduate Scholarships – Master's (CGS-M)
Walter H. Johns Graduate Fellowship Program
Alberta Graduate Excellence Scholarship (Indigenous) Program
Rupertsland Institute
Sustainability Scholar Program (the City of Edmonton and the University of Alberta)
ANID Fondecyt 11171006 and 1221528
Institute of Ecology and Biodiversity-Chile (Grant ANID/BASAL FB210006)
iCWild
National Research Foundation of South Africa
General Directorate of Nature Conservation and National Parks
European Food and Safety Authority
Noble Research Institute, LLC
Deutsche Forschungsgemeinschaft (DFG, German Research Foundation) under Germany's ExcellenceStrategy – EXC 2117 – 422037984
Technology Agency of the Czech Republic and the Ministry of the Environment within the "Prostředí pro život" Programme, No. SS05010008

Individual projects that contributed to this study received assistance from the following:

Wildlife Coexistence Lab, Faculty of Forestry, University of British Columbia
BC Parks
ACTo-SINAC
Murray State University Wildlife and Fisheries Society student members
Snapshot USA assistants

Lincoln Memorial students
M. Rossa, P. Linck and D. Hipólito
R. Bagchi of the University of Connecticut for help in data collection and design
Guillaume Morrel
University of North Dakota's (UND) Field Stations, Wildlife Society, and Wildlife Techniques Course
Levin Brandt
Wardens of Maremma Regional Park
R. Oliveira
University of Siena students
Wasatch Wildlife Watch community science team
Sageland Collaborative
Utah's Hogle Zoo
Salt Lake City's Trails and Natural Lands Division
University of Kansas Field Station
Harrison Stoudt
Elizabeth Saldo, Mike Muthersbaugh
Woodland Park Zoo community science volunteers and Seattle University students.
Georgia Department of Natural Resources - Urban Wildlife Program
Moritz Wenzler-Meya, Team 'Wildtierforscher' and Berlin Citizens
M. Bojda., M. Váňa, J. Labuda, B. Černá, K. Fridrochová, Ľ. Hrdý, J. Koranda and volunteers of "Wolf Patrols"
Marlin M. Dart
WildlifeACT ran the survey on Tembe Elephant Park, which was done with permission and support from Ezemvelo KwaZulu-Natal Wildlife. We would like to thank Sabi Sand Wildtuin and Mala Mala for allowing surveys of their reserves
Parks Canada staff, including Sarah Tyne, Daniel Grinnell, Michael Boudreau
Jon Stuart-Smith
Communities from the Amanã Reserve
McDowell Sonoran Conservancy stewards
KuzeyDoğa Derneği
Snapshot Wisconsin volunteers including Zooniverse photo classifiers
János Gombkötő
Mike D. Proctor and Derick Warren
oeko-log
Caylen Wolfer
Brandywine Zoo
Meet Your Wild Neighbours participants
Swedish University of Agricultural Sciences students
The Steering Committee of the COVID-19 Bio-Logging Initiative
Urban Wildlife Information Network (UWIN)
WildEdmonton volunteers and University of Alberta students
Disney Conservation Fund
Marine Ventures Foundation
Landsberg Foundation
Marin Fish and Wildlife

Fresno Chaffee Zoo
Conservation Society of California
Thornton S. Glide, Jr. and Katrina D. Glide Foundation
Olivia and Isabel Lepczyk
Ligue de Protection des Oiseaux France
