# Supplementary material for: Mammal responses to global changes in human activity vary by trophic group and landscape
Source: Nat Ecol Evol. 2024 Mar 18;8(5):924–35. doi: 10.1038/s41559-024-02363-2 (PMC11090811; doi:10.1038/s41559-024-02363-2)
Supplement: Supplementary file 2 — Reporting Summary [file 41559_2024_2363_MOESM2_ESM.pdf]

nature portfolio

Corresponding author(s): Cole Burton
Last updated by author(s): Jan 17, 2024

Reporting Summary

Nature Portfolio wishes to improve the reproducibility of the work that we publish. This form provides structure for consistency and transparency in reporting. For further information on Nature Portfolio policies, see our Editorial Policies and the Editorial Policy Checklist.

Statistics

For all statistical analyses, confirm that the following items are present in the figure legend, table legend, main text, or Methods section.

n/a	Confirmed	
☐	☒	The exact sample size (*n*) for each experimental group/condition, given as a discrete number and unit of measurement
☐	☒	A statement on whether measurements were taken from distinct samples or whether the same sample was measured repeatedly
☐	☒	The statistical test(s) used AND whether they are one- or two-sided<br>*Only common tests should be described solely by name; describe more complex techniques in the Methods section.*
☐	☒	A description of all covariates tested
☐	☒	A description of any assumptions or corrections, such as tests of normality and adjustment for multiple comparisons
☐	☒	A full description of the statistical parameters including central tendency (e.g. means) or other basic estimates (e.g. regression coefficient) AND variation (e.g. standard deviation) or associated estimates of uncertainty (e.g. confidence intervals)
☐	☒	For null hypothesis testing, the test statistic (e.g. *F*, *t*, *r*) with confidence intervals, effect sizes, degrees of freedom and *P* value noted<br>*Give P values as exact values whenever suitable.*
☒	☐	For Bayesian analysis, information on the choice of priors and Markov chain Monte Carlo settings
☐	☒	For hierarchical and complex designs, identification of the appropriate level for tests and full reporting of outcomes
☐	☒	Estimates of effect sizes (e.g. Cohen's *d*, Pearson's *r*), indicating how they were calculated

Our web collection on statistics for biologists contains articles on many of the points above.

Software and code

Policy information about availability of computer code

Data collection	No software was used to collect data
Data analysis	R Statistical Software, version 4.1.3. Code for data analysis are available on FigShare (link provided in Code Availability statement in paper).

For manuscripts utilizing custom algorithms or software that are central to the research but not yet described in published literature, software must be made available to editors and reviewers. We strongly encourage code deposition in a community repository (e.g. GitHub). See the Nature Portfolio guidelines for submitting code & software for further information.

Data

Policy information about availability of data

All manuscripts must include a data availability statement. This statement should provide the following information, where applicable:

- Accession codes, unique identifiers, or web links for publicly available datasets
- A description of any restrictions on data availability
- For clinical datasets or third party data, please ensure that the statement adheres to our policy

All data and code have been made available on FigShare with the links provided in the Data Availability and Code Availability statements in the paper.

Human research participants

Policy information about studies involving human research participants and Sex and Gender in Research.

Reporting on sex and gender	NA (our study did not involve human research participants)
Population characteristics	NA
Recruitment	NA
Ethics oversight	NA

Note that full information on the approval of the study protocol must also be provided in the manuscript.

Field-specific reporting

Please select the one below that is the best fit for your research. If you are not sure, read the appropriate sections before making your selection.

☐ Life sciences ☐ Behavioural & social sciences ☒ Ecological, evolutionary & environmental sciences

For a reference copy of the document with all sections, see nature.com/documents/nr-reporting-summary-flat.pdf

Ecological, evolutionary & environmental sciences study design

All studies must disclose on these points even when the disclosure is negative.

Study description	Comparison of amount and timing of animal activity between paired treatment (higher human activity) and control (lower human activity) time periods using detections from 5400 camera traps for 163 species across 102 survey areas. Unit of comparison was the population (species-project), with 1065 for amount of activity and 499 for timing of activity.
Research sample	Sample of terrestrial mammals detected at motion-triggered camera traps across 102 survey areas around the world. Surveys were identified opportunistically as those with active camera trap sampling before and during the COVID-19 lockdowns in 2020. Within surveys, cameras were deployed randomly or systematically to detect medium- and large-bodied terrestrial mammals (≥ 1 kg), including humans.
Sampling strategy	We included surveys for which: most or all camera trap stations were deployed in the same area of interest before and during COVID-19-related restrictions; a minimum of 7 unique camera trap deployment locations were sampled; a minimum sampling effort of at least 7 days per camera period; and trends in human detections were recorded from camera trap data or human activity was available from other sources.
Data collection	Mammals photographed by camera traps were identified from images by researchers from each project. The date and time of each detection was recorded, as was the location of each camera trap.
Timing and spatial scale	The spatial scale includes the entire world as we considered camera trap surveys from anywhere that met our criteria (listed above under sampling strategy). The timing of sampling varied by project, and across all projects spanned from 2017-2020, with most sampling between 2019-2020.
Data exclusions	We received data submissions from 146 projects, of which 112 met our sampling criteria (described above). We analyzed data from 102 projects, excluding 10 projects that did now show any change in human activity (i.e., no treatment effect).
Reproducibility	This was not a controlled experiment but rather a quasi-experiment based on changes in human activity in response to COVID-19 policies. Our samples were therefore not reproducible.
Randomization	Our comparisons of human and animal activity were between time periods within survey areas, thus controlling for variation between survey areas.
Blinding	Animals are detected by passive infrared cameras triggered by animal motion and body heat. There is no researcher bias in detecting animals.

Did the study involve field work? ☒ Yes ☐ No

March 2021

Field work, collection and transport

Field conditions	Our study covered 102 survey areas around the world, and compared animal detections between time periods. Field conditions thus varied substantially
Location	Our study covered 102 survey areas around the world.
Access & import/export	Camera trap sampling is non-invasive and does not involved any capture, handling or collection of animal specimens.
Disturbance	Camera trap sampling is non-invasive and causes minimal disturbance to animals.

Reporting for specific materials, systems and methods

We require information from authors about some types of materials, experimental systems and methods used in many studies. Here, indicate whether each material, system or method listed is relevant to your study. If you are not sure if a list item applies to your research, read the appropriate section before selecting a response.

Materials & experimental systems

n/a	Involved in the study	
☒	☐	Antibodies
☒	☐	Eukaryotic cell lines
☒	☐	Palaeontology and archaeology
☐	☒	Animals and other organisms
☒	☐	Clinical data
☒	☐	Dual use research of concern

Methods

n/a	Involved in the study	
☒	☐	ChIP-seq
☒	☐	Flow cytometry
☒	☐	MRI-based neuroimaging

Animals and other research organisms

Policy information about studies involving animals; ARRIVE guidelines recommended for reporting animal research, and Sex and Gender in Research

Laboratory animals	The study did not involve laboratory animals.
Wild animals	No animals were captured or handled. Animals were observed through photographic records obtained by passive infrared remotely triggered cameras
Reporting on sex	Both sexes for all species were recorded by photographs. There is no reason to expect any sex bias in sampling.
Field-collected samples	Study did not involve samples collected from the field (only photographs)
Ethics oversight	No ethical approval is required for non-invasive photographic sampling by passive, remote infrared cameras

Note that full information on the approval of the study protocol must also be provided in the manuscript.
